# How Intersectoral Priority Setting Can Support Action on Social Determinants of Health: A Qualitative Case Study From Pakistan’s Federal Health Ministry

**DOI:** 10.34172/ijhpm.9208

**Published:** 2026-07-01

**Authors:** Wafa Aftab, Ole F. Norheim, Inger Lise Teig, Sameen Siddiqi, Jesse B. Bump

**Affiliations:** ^1^Bergen Center for Ethics and Priority Setting in Health, Department of Global Public Health and Primary Care, University of Bergen, Bergen, Norway.; ^2^Department of Community Health Sciences, Aga Khan University, Karachi, Pakistan.; ^3^Takemi Program for International Health, Harvard T.H. Chan School of Public Health, Boston, MA, USA.; ^4^Department of Global Health and Population, Harvard T.H. Chan School of Public Health, Boston, MA, USA.

**Keywords:** Agenda Setting, Priority Setting, Social Determinants, Intersectoral Actions, Pakistan, Health Systems

## Abstract

**Background:**

Intersectoral actions can improve population health and reduce health inequities by addressing social determinants of health. Priority setting methods are used by health ministries to rationalize resource allocation within the sector, but not for intersectoral actions.

**Methods:**

We conducted a qualitative case study of an intersectoral priority setting exercise—a novel use of priority setting methods—undertaken by Pakistan’s Federal Ministry of Health between 2019–2022. Drawing on theories of agenda setting and priority setting, we examined the priority setting process through key informant interviews. We explored the perceived appropriateness of the priority setting approach and the challenges encountered during its implementation across sectors.

**Results:**

Our findings reveal that the process of intersectoral priority setting is deeply influenced by its political and institutional context. According to respondents, existing institutional structures and norms in Pakistan are not optimally suited to support intersectoral actions for health, although there is growing recognition of their importance. Regardless of sector, all respondents view engaging in intersectoral actions as part of the health ministry’s responsibility for population health. Priority setting, thereby, is viewed as a way of clarifying the health ministry’s intersectoral agenda. However, intersectoral priority setting methods are seen as underdeveloped in terms of available evidence, standardized economic and policy evaluation, and deliberative mechanisms. Respondents suggest that to implement the prioritized intersectoral agenda, the health ministry must effectively navigate the political space outside the health sector, such as by highlighting shared benefits, grounding the intersectoral agenda in local needs, and leveraging the political dynamics of other sectors. Moreover, this political engagement must be complemented by competence in evidence generation and communication to influence cross-sectoral policy dialogues.

**Conclusion:**

Using a priority setting approach, health ministries can systematically advance intersectoral health agendas by building political coalitions around shared priorities supported by rigorous evidence and analysis.

## Background

**Key Messages**

**Implications for policy makers**

- The choice of intersectoral interventions and associated determinants that the health ministry addresses is often ad hoc rather than based on a deliberate and systematic assessment of population health needs and optimal investment of resources.
- The health ministry could pursue intersectoral actions for health through a well-articulated and justified intersectoral agenda rather than as isolated and opportunistic actions.
- Intersectoral priority setting offers the health ministry a systematic approach for developing such an agenda for action. However, technical priority setting cannot be undertaken independently of the cross-sectoral political and institutional context, which determines the policy options available to the health ministry.
- An established intersectoral agenda for health could enable the health ministry to capitalize on intersectoral opportunities and promote a shared agenda across sectors within a broader human development framework. However, effectively fulfilling this role will require substantial strengthening of technical capacities within the ministry’s public health arm.

**Implications for the public**

Our study shows how health ministries can take a more systematic and proactive role in promoting health by prioritizing actions across other sectors, such as transport, environment, and education, that impact health outcomes. By using evidence-informed priority setting methods adapted for intersectoral decision-making, rather than ad hoc approaches, health policies can better address the root causes of illnesses such as pollution and poor infrastructure. Through political coalition-building based on sound analysis of important risk factors of illness, health ministries can influence broader policy-making. If applied widely, this approach could lead to more equitable and sustainable improvements in public health, particularly in low- and middle-income countries (LMICs) where health risks often lie outside the healthcare system. Ultimately, it offers a pathway to better health for all through more effective cross-sectoral decision-making for health.

Intersectoral actions, defined as the alignment of strategies and resources between two or more policy sectors to achieve complementary objectives,^1^ are crucial for improving population health and reducing health inequities by acting on determinants of health in collaboration with other sectors.^2^ Such interventions often require actions by non-health sectors yet produce considerable health benefits. For instance, expanding a public transport system based on clean energy improves air quality, reduces greenhouse gas emissions, increases physical activity, lowers obesity, and diminishes health disparities.3-5^3-5^ The World Health Organization’s (WHO’s) 2008 Commission on Social Determinants of Health recognized that such actions must involve multiple sectors but emphasized the central role of health ministries, saying that “*they can champion a social determinants of health approach…, demonstrate effectiveness through good practice…, and support other ministries in creating policies that promote health equity*.”^6^

 Such a role would require a health ministry to use its resources such as time, technical capacity and political capital to convince other sectors to act on those policies that protect or promote population health. However, health ministries in low- and middle-income countries (LMICs) often face constraints such as limited resources, pressing healthcare access demands, and external funding agendas, which may not be supportive of intersectoral work. Resources that health ministries can dedicate to intersectoral actions are thus limited and their effective prioritization especially pertinent. Health ministries in LMICs are increasingly using priority setting methods to design essential health services packages, to add rationality, transparency, and fairness to the use of resources.^7^ However, this approach has not been widely used for choosing priority intersectoral actions.

 We undertook a case study of an intersectoral priority setting exercise conducted by Pakistan’s Federal Ministry of National Health Services, Regulations and Coordination^[1]^ between 2019–2022 as part of the development of a universal health coverage package of essential health services in the country.^8,9^ The decision to include intersectoral interventions was based on the recognition that many risk factors such as tobacco use, physical inactivity, injuries, and air pollution cause a large share of ill-health but can only be addressed by policies outside the health sector.^10,11^ The process was led and coordinated by the health ministry with the involvement of relevant ministries such as the ministries of Planning and Development, Climate Change and Environmental Coordination, and Communication, among others. The multisectoral committee deliberated on and developed a set of intersectoral policy priorities based on the recommended intersectoral priority interventions in Disease Control Priorities 3 (DCP3).^10-12^ DCP is a World Bank publication series that identifies cost-effective interventions for the leading causes of disease burden in LMICs.^13^ The Committee appraised the 29 high priority intersectoral interventions recommended for LMICs in DCP3. Local perspectives and evidence were used to evaluate relevance for Pakistan, categorizing them into phased priorities for immediate, medium, and long-term implementation based on the current state of adoption and future feasibility. This multisectoral forum and its related processes were the focus of this study. The aim of the study was to explore whether priority setting could be a potentially useful approach for the health ministry to systematically address social determinants of health within local political and institutional context. Specifically, we explored stakeholders’ experiences with the perceived appropriateness of the priority setting approach for intersectoral actions and the challenges encountered during its implementation across sectors.

###  Theoretical Framework 

 Evidence-informed priority setting is a decision-making approach that informs how policy-makers could make the best use of scarce health resources. The aim is to determine how resources *ought* to be allocated for optimizing desirable outcomes such as longevity, quality of life, efficiency, and equity.^14,15^ Priority setting methods are increasingly used by health ministries in LMICs,^7^ to reduce ad-hoc decision-making, and to avoid implicit rationing that could hide unfair patterns in the distribution of healthcare resources.^16^

 In healthcare, priority setting is typically based on the cost-effectiveness of health interventions and other criteria, complemented by consultations among stakeholders.^17,18^ In its simplest form, a priority setting analysis would compare two drugs for the same condition but with different prices and efficacies to identify which one maximizes health within a fixed budget. This predominantly technical perspective does not capture critical aspects of intersectoral decision-making such as the political context that determines policy options and collaboration opportunities available to the health ministry.

 To understand how priority setting and its political context interact, we reviewed literature on agenda setting and priority setting theories to help us situate the priority setting role of the health ministry in an intersectoral political context. We searched for literature in Google Scholar using these search terms: agenda setting, framework, health, health policy, priority setting, resource allocation, deliberation, determinants of health, intersectoral, and multisectoral. No time limit was applied. Additional sources were identified via reference list screening of initial articles.

 We reviewed theories of agenda setting because they could provide insight into how various issues and policies acquire prominence in the political space making them viable policy options in the priority setting process for health and other ministries. This link between priority setting and political agenda setting theories has been explored only rarely,^19,20^ possibly because of their divergent disciplinary backgrounds and epistemic traditions. Priority setting is based on a rationalist, positivist perspective, influenced by economic methods, and is normative in character.^21^ Agenda setting theories are grounded in social constructivism, draw on theories of political economy and are often descriptive rather than normative.^21,22^ However, for intersectoral actions, examining the interaction between priority setting and agenda setting could identify opportunities for action that might not emerge when either approach is used alone.

###  Frameworks of Priority Setting

 To understand how priority setting constructs apply to intersectoral interventions we searched the literature on priority setting. We identified several frameworks for priority setting of health interventions but none that addressed intersectoral actions for health specifically. Of the identified frameworks, we included three in the analysis: Multicriteria decision analysis (MCDA), Accountability for Reasonableness, and evidence-informed deliberative process. They were chosen because they are applicable to health and non-health interventions, are widely used, and are generalizable rather than specific to a context. Table 1 presents the key elements of each framework and synthesized elements relevant to intersectoral priority setting.

**Table 1:** Key Elements of Selected Frameworks of Priority Setting and Synthesized Elements Relevant to Intersectoral Priority Setting

**Key Element of Frameworks of Priority Setting**			**Synthesized Themes Relevant to Intersectoral Priority Setting**
**MCDA**	**Accountability for Reasonableness **	**Evidence-Informed Deliberative Process **	
Defining the decision problem Selecting and structuring criteria Measuring performance Scoring alternatives Weighting criteria Calculating aggregate scores Dealing with uncertainty Reporting and examination of findings	Relevance Publicity Revision and appeal Regulative condition	Situational analysis Formation of stakeholder panel Identification of relevant criteria Collection of evidence on criteria Deliberation Recommendations for implementation	Defining the scope of the problem Choice of stakeholders Choice of substantive criteria to guide policy choice Collection of evidence and evaluation through deliberation Policy recommendations Mechanisms for repeal and review

Abbreviation: MCDA, multicriteria decision analysis.

 MCDA is a decision-making framework commonly used in many policy domains^23,24^ including health.^25^ The most commonly used quantitative iteration of MCDA, based on decision theory and multi-attribute utility theory, allows optimization for more than one policy objective,^26^ and is thus suitable for intersectoral policies with health and non-health effects. MCDA begins with clarifying the scope of a decision problem including its objectives, alternatives, and stakeholders.^27^ Subsequently, criteria for guiding the decision are chosen, and evidence is collected and collated into a performance matrix. This is followed by scoring, weighting and aggregating scores, or a qualitative ranking of options.^16,27^ The findings are then reported to policy-makers who use this information to choose policy options.

 Intersectoral interventions require input of multiple stakeholders from different sectors, however, MCDA does not specify the dynamics of such multistakeholder decision-making. To capture these deliberative aspects of decision-making, we considered the Accountability for Reasonableness framework. Daniels and Sabin^28^ proposed this framework to enhance the fairness and legitimacy of decision-making processes in health. They reasoned that if the process was considered fair, then resulting decisions would be accepted even by those adversely affected. Such a decision-making process must fulfill four conditions: first, the grounds on which decisions are made should be publicly accessible; second, the use of scarce resources should be justified by evidence, argument, and reasons; third, mechanisms should exist for appealing the decision and for revising them should new evidence become available; and finally, the above three conditions must be enforced.^28^

 Both frameworks have been widely used in resource allocation decisions. MCDA has been used to provide a transparent substantive basis for making resource allocation decisions to enhance public acceptance of rationing decisions.^29-31^ Accountability for Reasonableness is the most influential procedural framework in health promoting the use of fair processes to enhance legitimacy of priority setting decisions.^32-34^ However, the two frameworks have been critiqued on different grounds: Accountability for Reasonableness for not offering specific decision-making criteria,^35^ and MCDA for giving insufficient attention to stakeholders.^36^ Combining substantive elements of MCDA with deliberative aspects of Accountability for Reasonableness has been proposed as a logical way of overcoming these shortcomings.^36^

 Baltussen et al have combined these substantive and procedural aspects in the Evidence Informed Deliberative Process framework.^37^ Their framework requires the use of evidence such as on the costs and effectiveness of interventions and the use of explicit criteria (such as equity and feasibility). They acknowledge that evidence needs to be evaluated and interpreted because priority setting is a political process laden with differing values and interests. Their framework thus grounds evidence within a deliberative process recognizing that reasonable people might disagree about relevant principles and how they ought to be balanced. This framework systematizes these elements in a six-step process of priority setting: situational analysis, formation of stakeholder panels, identification of relevant criteria, collection of evidence, deliberation among stakeholders, and recommendations for implementation.

###  Theories of Agenda Setting

 Frameworks of priority setting in health largely do not examine the politics of priority setting, even though some frameworks acknowledge the importance of doing so.^37^ Intersectoral policies are particularly influenced by broader political processes and separating intersectoral priority setting from its political context might limit its relevance. Since we could not find any theoretical frameworks focused on the politics of priority setting within health or other sectors, we looked to the literature on the politics of agenda setting. We reasoned that theories of agenda setting would be applicable to our case because of two interconnected reasons. Enacting intersectoral policies is often the mandate of non-health sectors. Their policy objectives and agendas might differ from the health sector, and health actors may have to act within those boundaries or negotiate them. Moreover, different sectors have potentially conflicting agendas, varying levels of power and access to resources that they can leverage to get issues of interest on the policy agenda. Agenda setting theories, focusing on power and how it shapes policy choices, are therefore useful to inform this discussion. Furthermore, generating and sustaining action on these policies requires collaboration and alliance formation, which some agenda-setting theories help us understand.

 We selected four agenda-setting theories because of their frequent application to health policies as well as broader applicability to intersectoral decisions focusing on multiple stakeholders, institutional influences, and the interaction of these factors with evidence-informed policy solutions. These four theories are: Hall’s model of legitimacy, feasibility and support; Mills’ power elite theory; Kingdon’s three streams model; and Hall’s 3i model. A summary of the elements of each theory and a synthesis relevant to intersectoral priority setting is presented in Table 2.

**Table 2:** A Summary of the Elements of Selected Theories of Agenda Setting and Synthesized Elements Relevant to Intersectoral Priority Setting

**Key Element of Theories of Agenda Setting**				**Synthesized Themes Relevant to Intersectoral Priority Setting**
**Kingdon **	**3i**	**Hall **	**Power Elite **	
Problem stream Policy stream Politics stream Windows of opportunities and the role of policy entrepreneurs	Ideas Institutions Interests	Legitimacy Feasibility Support	Various interests of elite groups Distribution of power among elites Interaction of power groups Conditions for alliances	The perception of the intersectoral problem by government, the public and elite groups as an issue ought to be addressed by the state Institutional roles and willingness and incentives to collaborate Availability of feasible policy solutions to address the problem Windows of opportunity for action

 Hall’s model proposed that an issue is likely to reach the policy agenda when it is high in legitimacy, feasibility, and support.^38^ *Legitimacy* refers to the government’s belief that it should intervene in an issue; *feasibility*, to its ability to implement the policy given resource requirements, whether technical, monetary, human resource, administrative, or infrastructure; and *support*, to the level of public backing for the government concerning the issue in question.^22,38^ However, leveraging action on intersectoral policies is dependent on a broader set of actors within and outside the government than Hall presumes, and not all actors possess equal power to influence policy agendas. The power elite theory proposed by Mills posits that power is disproportionately distributed in society. A power elite consisting of powerful individuals and groups use their power to align policy agendas with their interests.^39^ However, agenda setting is dynamic, and there is a temporal dimension of agenda setting that neither framework captures. Kingdon’s three streams theory proposes that three separate streams of problems, politics, and policies influence whether issues rise on the policy agenda.^40^ The three streams normally function independently, but issues ascend on the policy agenda when the streams come together to create ‘windows of opportunity’ when decision-makers are particularly receptive to an issue.^40^ The 3i framework proposes that policies are determined by the interests and ideas of policy actors, mediated by institutions.^41^ Here, *ideas* are the beliefs about what is or ought to be and include knowledge and values;* interests* represent the agendas of different stakeholders; and *Institutions* are the ‘rules of the game’ that determine how various actors interact with each other.^42^ Institutions include both formal structures and procedures as well as informal norms of behavior.^22^

## Methods

###  Study Design

 The intersectoral priority setting process led by Pakistan’s Federal Health Ministry offered a unique opportunity to study this novel application of priority setting methods to intersectoral actions for health. To explore this, we conducted a qualitative case study using key informant interviews. The case study approach enabled a contextual understanding of the phenomena in question.^43^ Moreover, semi-structured interviews can provide in-depth insights into the experiences and reasonings of those involved in the priority setting process.^44^ We wanted to explore the perceived appropriateness of the priority setting approach and the challenges encountered during its implementation across sectors. We conducted semi-structured interviews with 14 key informants (Table 3). Initially, we used purposive sampling to select participants directly involved in the intersectoral priority setting process, focusing on actors from health and non-health sectors closely involved in the process. Supplementary snowball sampling allowed us to reach additional informants with relevant expertise, including those who, based on their familiarity with the health and intersectoral policy-making landscape in Pakistan, provided valuable institutional and political insights.

**Table 3:** Profile of Key Informants

**Institution **	**Number of Key Informants **
Health sector	
Health (policy unit in the ministry of health)	2
Health (directorate)	2
Health minister (former)	1
Nutrition	1
Other sectors	
Ministry of Planning and Development/Planning Commission	3
Ministry of Communication (and transport)	1
Ministry of Climate Change and Environmental Coordination/Environment Protection Agency	1
Cabinet division expert	1
Non-government experts	
Public policy expert	1
Public health expert	1
Total	14

###  Data Collection

 We developed a semi-structured interview guide whose key domains are shown in Box 1. We gathered the responses of 14 individuals in 13 interviews between June and August 2023. Interviews were conducted in Urdu. Three interviews were conducted online via Zoom while the rest were conducted in-person. All interviews were audio-recorded. Informed written consent was obtained from all key informants at the start of the interview. At the end of each interview, informants were asked to suggest the names of other potential respondents. Recruitment ended when we judged that saturation had been reached, which happened sooner for the process of priority setting but required more interviews for the political context.

**Box 1. Key Domains in the Interview Guide**

1. What were the impressions and experiences of the priority setting process? Was it useful? *[criteria, deliberation, platforms]*
2. [For people involved in the development of essential health benefit package as well as intersectoral interventions for health] how did the experience of choosing priority intersectoral interventions differ from the experience of choosing healthcare interventions?
3. Where should the overall process be based and who should lead it?
4. What should be the role of the health ministry vis-à-vis other sectors in this process?
5. What sort of information/evidence was used to choose policies? On what basis should the policies be chosen? How could different criteria be applied?
6. The culture and mechanisms of decision-making in health and other sectors – whether and how they differ. And how should those differences be accommodated in priority setting processes for intersectoral actions?

###  Data Transcription and Analysis

 The recordings were translated into English and transcribed. Data were secured in password-protected files and anonymized before analysis. We conducted a reflexive thematic analysis which offered a flexible approach involving inductive and deductive analysis as well as theory- and data-driven analysis conducted within an interpretivist paradigm.^45,46^ The initial analytic phase was inductive and data-driven to ensure that participants’ accounts shaped the early coding process rather than being constrained by prior theoretical assumptions. During this initial phase, we generated codes that captured patterns related to how priority-setting methods were conceptualized and justified, as well as the broader political context in which these methods operated. As the analysis progressed, we introduced relevant theoretical perspectives to support a more interpretive and conceptually informed exploration of the data. This allowed us to refine and consolidate the inductive codes into themes that addressed our research questions. The development of these themes was informed by theories of priority setting and agenda setting.

## Results

###  Theme 1: Policy Agenda and Intersectoral Policies for Health

####  Low Legitimacy and Support for Preventive Policies for Health 

 All health respondents said that the health policy agenda remains concerned with curative rather than preventive actions. This curative emphasis was attributed to the higher visibility of curative services, rendering them a priority for policy-makers. According to respondents, this tendency is partly driven by the higher demand for curative, rather than preventive, services among funding agencies and the public. Respondents said this was one reason health ministries^[2]^ persistently prioritize healthcare services rather than addressing determinants of health. As one respondent explains:

 “*In the case of (intersectoral) health interventions, especially preventive health interventions, policy-makers, the public, politicians, bureaucrats—they are simply unable to understand why they should spend money on this” *[Public policy expert].

 One respondent cited air pollution to illustrate an issue with serious health consequences that is not considered a health issue by the public or policy-makers except in the most directly linked conditions, such as respiratory illnesses. Conceived in this way, the prevention of air pollution is far less relevant to the health sector than if its broader effects were appreciated, including diabetes, cognitive impairment, stroke, ischemic heart disease, chronic obstructive pulmonary disease, reproductive issues, and more.

 Many respondents pointed to systemic institutional factors within health ministries as reasons for neglecting preventive actions in health policy agenda. When talking about the policy-making process, most respondents portrayed a decision-making culture that is reactive rather than proactive. They described a bias towards urgent and immediate problems, precluding focus on long-term and preventive solutions. Respondents pointed out that short, unpredictable tenures and frequent turnover in health leadership foster an environment that favors short-term decision-making over long-term mitigation of health risks. Thus, without widespread support, and due to a lack of preventive orientation in policy-making, intersectoral actions for health are either perceived as beyond the legitimate purview of the health ministry or not given priority.

####  Intersectoral Actions for Health Gain Policy Priority Only Under Certain Conditions

 Many respondents pointed out that effective intersectoral action for health tends to occur only under particular circumstances. They described that decision-makers across different sectors are more willing to engage in intersectoral actions during emergencies such as floods or a Dengue outbreak; instances of heightened media attention, when the issue becomes a high-level political priority; or if there are significant economic consequences. As one respondent described:

 “*In our system, cross-sectoral collaboration only occurs if it starts in a high office and then trickles down, becomes a news headline, or there are considerable financial or economic implications, whether negative or positive” *[Public policy expert].

 Many respondents readily pointed out COVID-19 as an event that prompted effective intersectoral actions. They explained that in the case of COVID-19, a sense of urgency, strong media focus, political priority at the highest levels of the state exercised through strong executive oversight and support from the powerful military, and potentially severe economic consequences converged to create an environment that enabled rapid and effective collaboration across ministries of health, interior, planning, education, labor, among others. Some respondents, however, clarified that the intersectoral response to the pandemic arose out of a unique set of circumstances and does not reflect a willingness to engage in routine intersectoral actions such as those aimed at addressing determinants of health. Others noted that such a convergence of favorable conditions is improbable for less conspicuous issues, such as air pollution or climate change.

 These views imply that though an intersectoral response to health risks perceived as urgent can be mounted rapidly and effectively, less visible long-term risks need to be proactively and strategically highlighted to gain prominence in policy agendas.

###  Theme 2: Institutional Mechanisms, Mandates, and Norms for Intersectoral Actions for Health

####  Growing Recognition of the Need for Intersectoral Actions but Limited Institutional Mechanisms 

 Respondents indicated that policy-makers are becoming more aware of the necessity for intersectoral actions to solve complex policy issues that span multiple sectors. They attributed this increased awareness to the cross-linkages highlighted by Sustainable Development Goals (SDGs), emphasis on intersectoral actions by multilateral agencies and donors, and the government’s efforts to achieve multiple objectives from policies to overcome fiscal constraints across sectors.

 One respondent considered intersectoral decision-making to be the norm at the highest executive and legislative forums. In their view, several forums such as cabinet committees and Parliamentary standing committees consider issues in a way that transcends sectoral boundaries.

 In contrast, respondents from several departments said that existing institutional structures do not support collaborative decision-making and implementation. Respondents from the ministries of health, environment, and transport reported a lack of clear mechanisms for intersectoral work, which, in their opinion, typically occurs only on a case-by-case basis. Respondents mentioned the existence of ad hoc mechanisms for multi-stakeholder issues eg, a committee for international health regulations, but noted that formal, routine mechanisms for cross-sectoral collaboration do not exist. As noted by a former federal health minister, although the need for intersectoral actions is recognized, effective implementation mechanisms remain lacking.

 “*The issue is not of understanding but of implementation or management. Means are not there, forums are not there, mandates do not exist” *[Respondent health sector].

 Contradicting this view, respondents from the Planning Commission disputed the notion of ambiguous institutional roles, saying that intersectoral collaboration is clearly the Commission’s mandate based on the government’s formal Rules of Business. They explained that any issues outside the mandate of specific ministries fall under the Planning Commission’s purview, which exercises its convening authority to bring different ministries together on a common platform.

 “*…we have very clear rules of business for every ministry, department and the Planning Commission. No one (else) has the role of multisectoral coordination and the mandate for convening across sectors, except for the Planning and Development Department and the Planning Ministry”* [Respondent Federal Planning Commission].

 Stakeholders from health, transport, and environment acknowledged the Planning Commission’s role but were more concerned with the effectiveness of this mechanism rather than the existence of a formal mandate. In their view, the Commission does not allow genuine intersectoral work since its rules allow each policy proposal to be ‘owned’ only by one sector precluding formally shared decision-making, implementation, or accountability.

 Overall, respondents involved in the priority setting process whether from health or other sectors conceived priority setting and implementation of intersectoral interventions as interconnected rather than discrete processes. Consequently, the perceived lack of institutional mechanisms and incentives for collaboration led many to view priority setting without supportive institutional structures, norms and political will as inconsequential.

####  Overcoming Siloes Is Challenging Even in Intersectoral Forums 

 Many respondents noted that the strictly sectoral nature of institutional roles was not the only systemic barrier to effective collaboration. They noted that different sections, even within the health ministry, often operate in isolation, reflecting a culture of siloed working and limited coordination. Health respondents found that collaboration is easier with some ministries than others and that following formal rules for approaching other ministries does not always result in a willingness to collaborate. One health respondent suggested that power imbalances between ministries might be an underlying reason. For instance, with significant funding and a prominent global agenda, the Ministry of Climate Change was perceived to be less willing to engage on equal terms. In contrast, other ministries such as agriculture were seen as more approachable owing to established linkages through existing forums. Other respondents talked about the difficulty of changing norms that militated against working across sectoral lines even when supportive institutional structures were created.

 “*…e.g., our (Planning Commission’s) SDG (unit),… coordination was also minimal… So, when a project is made for an initiative like this, that also goes into a silo” *[Respondent Federal Planning Commission].

 Overall, respondents’ views imply that shared decision-making and implementation is not simply a matter of creating new cross-sectoral institutional structures but includes the more challenging task of shifting norms and incentives towards collaboration.

###  Theme 3: The Health Ministry’s Role in Addressing Health Determinants Across Multiple Sectors 

####  The Health Ministry’s Responsibility Extends Beyond Health Service Delivery to Determinants of Health

 All health and some non-health respondents considered it necessary to work across different sectors to improve population health. All health respondents envisioned the health ministry’s role as extending beyond mere health service delivery to a broader responsibility for population health including social determinants.

 However, health respondents also agreed that the health ministry has not meaningfully integrated intersectoral actions or social determinants in their work, relying instead on isolated and opportunistic initiatives driven by the personal interests of individual ministers or bureaucrats.

 Several non-health respondents concurred with this extended responsibility of the health ministry and described how the health sector can shape non-health policies that affect population health. For instance, a respondent from the transport sector said that it was in the health sector’s interest to coordinate in specifying ways of making road infrastructure and regulations safer given the high health toll of road traffic incidents.

 Many respondents thus consider it legitimate for the health sector to participate in those non-health policy decisions that affect population health. Health sector respondents expressed stronger views framing this as imperative for the health ministry while acknowledging that this responsibility is not currently systematically executed.

####  Health Ministries Must Learn to Navigate the Political Space Beyond the Health Sector

 Some health, as well as non-health respondents, believed that practically fulfilling this extended responsibility for population health requires the health ministry to operate effectively in the political space outside the health sector.

 Health ministry respondents sought clarity on implementing intersectoral priorities, feeling constrained by institutional structures but also recognizing existing opportunities. For example, they mentioned creating one-on-one relationships with other sectors to share resources and leverage good relationships. A health respondent recommended involving decision-makers from other sectors in internal discussions to build a more receptive audience for policy proposals. They gave the example of inviting a decision-maker from the Federal Bureau of Revenue to discussions about tobacco taxation before sending a formal policy proposal through the Ministry of Finance.

 Others, however, thought that the ministry should take a much more systematic approach. At the higher political level, some health respondents thought that the health sector must cultivate a shared agenda for addressing social determinants across different sectors by positioning health within a broader human development framework rather than focusing only on health objectives.

 “*I would rather frame them (intersectoral actions for health) in a human development or social development framework because then you can talk about shared goals, shared responsibility, a shared vision. If health goes and says to the environment ministry: Health is being damaged, you should sort this out, work with us, and we will coordinate with you. That’s an entirely different approach than looking at it from a slightly higher level, (which is that) we are all (ministries) working for national development. I think it is very important to promote the idea of a shared responsibility” *[Respondent health sector].

 Other respondents highlighted the role of political leadership in navigating the cross-sectoral space. One non-health respondent said that to successfully engage other sectors health ministry must understand how other sectors operate, identify those who influence policy decisions, locate the barriers to policy uptake and determine how to bypass them. Non-health respondents also said that it was important to intervene at the right point in the internal policy cycle of other sectors such as when rules and regulations are being developed rather than trying to change established policies. Highlighting the role of high-level political advocacy, one non-health respondent said:

 “*...a strong health minister can certainly bring a lot of change…ministers with a strong portfolio usually have a say. People respond to them because they have a reputation based on their work” *[Respondent Environment Protection Agency].

 Thus, while there was some divergence among respondents about ways of engaging with non-health actors, the need for such engagement was unanimously felt. Respondents in technical roles in the health ministry tended to favor issue-based engagement possibly linked to their own decision space. However, political leaders in the health sector and many non-health respondents suggested a more strategic approach from political leadership in the health sector. This approach was characterized as developing shared agendas across sectors, understanding internal political dynamics of other sectors, and forging alliances with leaders and policy entrepreneurs in other sectors.

####  Limited Technical Capacity in the Health Ministry Hinders Effective Intersectoral Participation 

 While many respondents saw the priority setting process as potentially useful, they thought that to articulate and implement this agenda across sectors, health ministries must develop necessary technical capacities. The consensus among respondents was that the health ministry currently lacks the required technical expertise.

 Respondents noted that the health ministry’s technical wing lacks skills and is disempowered. In their opinion, decision-making and policy direction are driven by non-technical civil servants, sidelining the technical staff. Health directorates, meant to play a technical role, are reduced to executing decisions made by a politically powerful but non-technical bureaucracy.

 Non-health respondents also talked about a dearth of technical expertise in health when called for, hence their reliance on consultants and experts. They emphasized the crucial role of well-resourced public health institutions as the health ministry’s technical arms, essential for developing evidence to advocate for health-relevant intersectoral actions.

 “*… such institutions [public health institutes] are doing this work all over the world and providing advisories on these matters. When we need advice, we hire a consultant or constitute a committee but largely we do not have an institution like this. That is why when we need any technical assistance, we engage experts or consultants” *[Respondent Federal Planning Commission].

 This respondent further said that the supportive role of public health institutes should include producing, collating and interpreting evidence and presenting it in accessible formats for policy-makers in other sectors. A transport sector respondent cited the use of health facility catchment data to identify locations requiring infrastructure improvements, reducing fatalities and disabilities. A respondent from the Environmental Protection Agency noted that data linking their activities to health outcomes could motivate their leadership.

 These views suggest that political engagement with other sectors must be complemented by technical competence for informing cross-sectoral policy debates. Encouragingly, there is an existing demand in some intersectoral decision-making forums for technical input from the health sector. However, the health ministry seems unable to capitalize on this demand due to insufficient technical skills in evidence generation and communication.

###  Theme 4: Intersectoral Priority Setting Approach Is Useful but Underdeveloped 

####  Local Health Needs and Well-Articulated Objectives Should Guide Intersectoral Priority Setting 

 While higher-level policy-makers viewed the priority setting process as an opportunity for the Ministry of Health to define its agenda on social determinants of health, other stakeholders highlighted diverse reasons for engaging in the process. Several health and non-health respondents stated that the priority setting process offered an opportunity to develop a shared understanding of intersectoral policies. Highlighting the lack of accountability in intersectoral actions, a respondent from the transport ministry viewed the process as a potential venue for ongoing shared monitoring. Others saw the value of priority setting as an ongoing dialogue, rather than a one-off exercise, to foster partnerships and enhance collaboration and implementation. Stakeholders from other sectors viewed the process as an opportunity to engage with health actors and link their policies to their health outcomes, especially in the absence of other forums for such discussions.

 Some respondents questioned the usefulness of a process which, to them, seemed disconnected from local needs. They thought that instead of being based on a predetermined package such as from DCP3, priority setting should be based on an assessment of local needs. An alternative approach recommended by some respondents was to understand the nature of local risks to population health as the starting point guiding the choice of interventions. As one respondent said:

 “*… in our context, what are our needs currently. What gaps need to be addressed? And based on that what should be our package that conforms to the aspirations of all jurisdictions of this country. First, there should have been a needs assessment; a survey of the existing situation and gaps. And discussions should have been based on that information. In my view, ideally, that’s what should have happened”* [Respondent Federal Planning Commission].

 In this respondent’s view, priority setting should be grounded in local needs rather than a pre-developed generic package of interventions for LMICs. Concurring with this view, another respondent said that using a pre-determined list of potential interventions, rather than open-ended discussions at the beginning, gave the impression that key decisions about policy choices had already been made prior to consultation.

 Considerable perceived differences in the purpose of intersectoral priority setting suggest that, at the outset, a shared understanding of objectives, meaningful for all stakeholders, may need to be developed. Moreover, for priority setting to be considered consequential for public policy, proposed policy options must be demonstrably grounded in the local context. Meaningful stakeholder engagement might require that they be involved throughout the development of policy options and that there is transparency about the reasons for policy choices under consideration. Otherwise, there is a risk that deliberation might be seen as a box-ticking exercise rather than shared decision-making, obstructing commitment to resulting decisions.

####  Intersectoral Priority Setting Methods Need to Be Developed Beyond Those for Health Interventions 

 Respondents highlighted several factors that made intersectoral priority setting uniquely challenging. Several respondents mentioned the difficulty of finding evidence of the effectiveness of intersectoral actions, particularly in Pakistan’s context, limiting the extent to which priority setting could be informed by evidence. They contrasted this with health interventions where evidence about effectiveness and cost-effectiveness was readily available.

 Moreover, they mentioned that the effectiveness of intersectoral interventions is reported in a variety of metrics from benefit-cost ratios, and net benefits to disability-adjusted life years. The lack of standard metrics meant that they could not rank these interventions as easily as health interventions based on incremental cost-effectiveness ratios. Similarly, while several respondents mentioned that equity impact was taken into consideration, there were differing views on the extent to which this was successfully done citing a lack of information and standard methods to assess and compare equity impact.

 In comparing the deliberative processes for the two packages, respondents highlighted the challenges involved in engaging other sectors, which they found to be more difficult than anticipated. Some respondents linked this difficulty to the lack of an overarching cross-sectoral forum to affirm the final intersectoral package. They mentioned that the interministerial forum responsible for approving both packages consisted only of federal and provincial health ministries, but such decisions require a cross-sectoral forum to give the health sector a formal mandate to engage other sectors.

 Given these challenges, one health respondent said about the priority setting process:

 “*The decision-making process was really hampered by lack of evidence and our lack of confidence in taking decisions… it reflects our lack of confidence in the deliberative process because of the lack of evidence” *[Respondent Health sector].

 Overall, these responses signal the need for development of robust methods for intersectoral priority setting for health before health ministries can reliably use this approach. Respondents’ views suggest that rather than being treated as an extension of the health benefit package, intersectoral priority setting should be based on specific approaches such as appropriately empowered decision-making forums which lend it legitimacy beyond the health sector, ensuring representation of key non-health actors, and the need for relevant evidence in appropriate formats.

## Discussion

 To our knowledge, this is the first study that examines a systematic application of priority setting methods to intersectoral interventions for health in an LMIC. Our findings underscore the intrinsic connection between intersectoral priority setting and its political and institutional context. We found that existing institutional structures in Pakistan are not optimally positioned for intersectoral actions for health. Health ministries can effectively foster intersectoral actions by devising their own prioritized policy agenda and developing consensus on it across sectors. However, this requires enhanced technical capacities in the health ministry and public health institutions, identified as one investment avenue for promoting action on social determinants of health. Additionally, health ministries must engage effectively with the broader political landscape to steer this prioritized agenda across sectors. Finally, several methodological and normative challenges about intersectoral priority setting remain to be resolved (Supplementary file 1, Table S1).

 Many respondents found intersectoral priority setting to be useful but highlighted several challenges. Those who were closely involved thought that intersectoral priority setting required a different approach than priority setting of health interventions. Conceptually, priority setting in health is a problem of allocating health sector resources to improve health outcomes.^47^ But for intersectoral interventions there are multiple outcomes and several budget holders with potentially differing priorities. Even if the health ministry developed its own prioritized agenda for intersectoral actions, it must actively shape policy responses in cross-sectoral platforms. Moreover, the health ministry might face constraints such as insufficient technical capacity and limited ability to persuade other sectors to act given the effort, time and political competence required.^48,49^ Thus, the politics of intersectoral priority setting are even more complex than that involved in priority setting within the health sector.

 While health respondents expected intersectoral priority setting to be complex, they seemed to have underestimated the political and institutional challenges. De Leeuw refers to a certain naivete among health advocates about the profound role of power and politics in the intersectoral arena ascribing it to the ‘moral high ground’ that many in the health sector assume about health.^50^ Priority setting, if conducted as a merely technical process, removes this link between politics and policy-making, leaving health stakeholders unprepared for engaging with the political aspects of cross-sectoral work. Health actors, therefore, must recognize that health is one among many policy objectives, and it is not immune to the bargaining and negotiation that is a routine part of the political process.^51^ For intersectoral priority setting, this presents a two-fold challenge: the methodological challenge of clarifying and measuring multiple outcomes and trade-offs and the complex political task of negotiating them. Respondents highlighted several unresolved normative and methodological challenges in intersectoral priority setting such as identifying relevant criteria like equity, feasibility, and efficiency, and aligning them with shared goals beyond health. Another key issue is identifying and accessing the types of evidence needed to inform decisions across sectors.

 The overriding opinion among our respondents was that a strictly sectoral institutional make-up prevented effective intersectoral collaboration. While institutions influence policy-making dynamics, recent theories of institutionalism^52^ caution against ascribing excessive explanatory power to them. According to this account, institutions do not determine policies but are merely one of the factors that affect them. Furthermore, institutions are not immutable. They are the result of certain power structures and policy choices and can be renegotiated based on extra-institutional political and ideational conditions,when dissatisfaction with existing institutions increases.^52^

 However, the health ministry may find it difficult to achieve significant institutional change on its own. Some respondents suggested that an alternate pathway was to develop collaboration with other sectors based on shared goals. Recent literature on intersectoral collaboration for health supports this view. Greer et al propose a *health for all policies* approach that focuses on co-benefits as the basis of intersectoral collaboration, rather than the *health in all policies* approach which relies solely on convincing other sectors to include health impact in policy evaluations.^53^ Greer et al argue that given the proven inability of health actors to compel other sectors to prioritize health, a ‘win-win approach’ focusing on co-benefits and fostering coalitional politics could be more effective, indicating an interplay between technical evaluation and the broader political context.

 To play this collaborative role effectively, health ministry and public health institutions must be equipped with the requisite capacities and resources. Leppo et al^54^ argue that the health ministry must play the dual role of generating policy-relevant evidence as well as managing the politics of divergent interests and power relations to promote intersectoral actions for health. They recommend that health ministries must develop the skills to generate evidence, translate evidence into policy, develop consensus across sectors for priority policies, and support effective implementation. They contend that generating evidence on health and health equity impact of policies of other sectors is an essential skill for the ministry of health.

 Our findings also suggest that the health ministry must intervene in the policy process at an opportune time when the political process is more receptive to change such as when policies are being drafted. Being prepared to intervene in its priority areas would also allow the health sector to take advantage of ‘windows of opportunities,’ which may not last indefinitely and require expedient action.^40,54^ Having clear priorities would also allow health ministries to play an active role in creating opportunities for action rather than merely reacting. Table S2 (Supplementary file 1) links our findings to the agenda setting domains of the framework and their implications for health actors.

 While existing literature recognizes that politics influences intersectoral priority setting,^12^ limited attention has been paid to how political processes interact with the technical stages of priority setting and how these domains may mutually reinforce or constrain one another. Figure illustrates these interactions, inferred from our empirical findings and informed by the theoretical framework. The figure shows that the policy options available to health actors (“windows of opportunity”) at any point are shaped by a dynamic political environment. This environment influences which priorities are feasible, legitimate, or strategically advantageous and therefore should inform deliberations in the priority setting process. Moreover, the figure clarifies the political factors that may be amenable to modification when a health ministry wants to make specific priorities more prominent (“catalyzing action”), such as providing evidence to raise support and legitimacy, identifying shared incentives across sectors, and increasing political and technical feasibility by producing evidence on expected intervention effects across health and non-health outcomes. Together, these stages illustrate how political and technical considerations intertwine to shape intersectoral priority setting.

**Figure:**
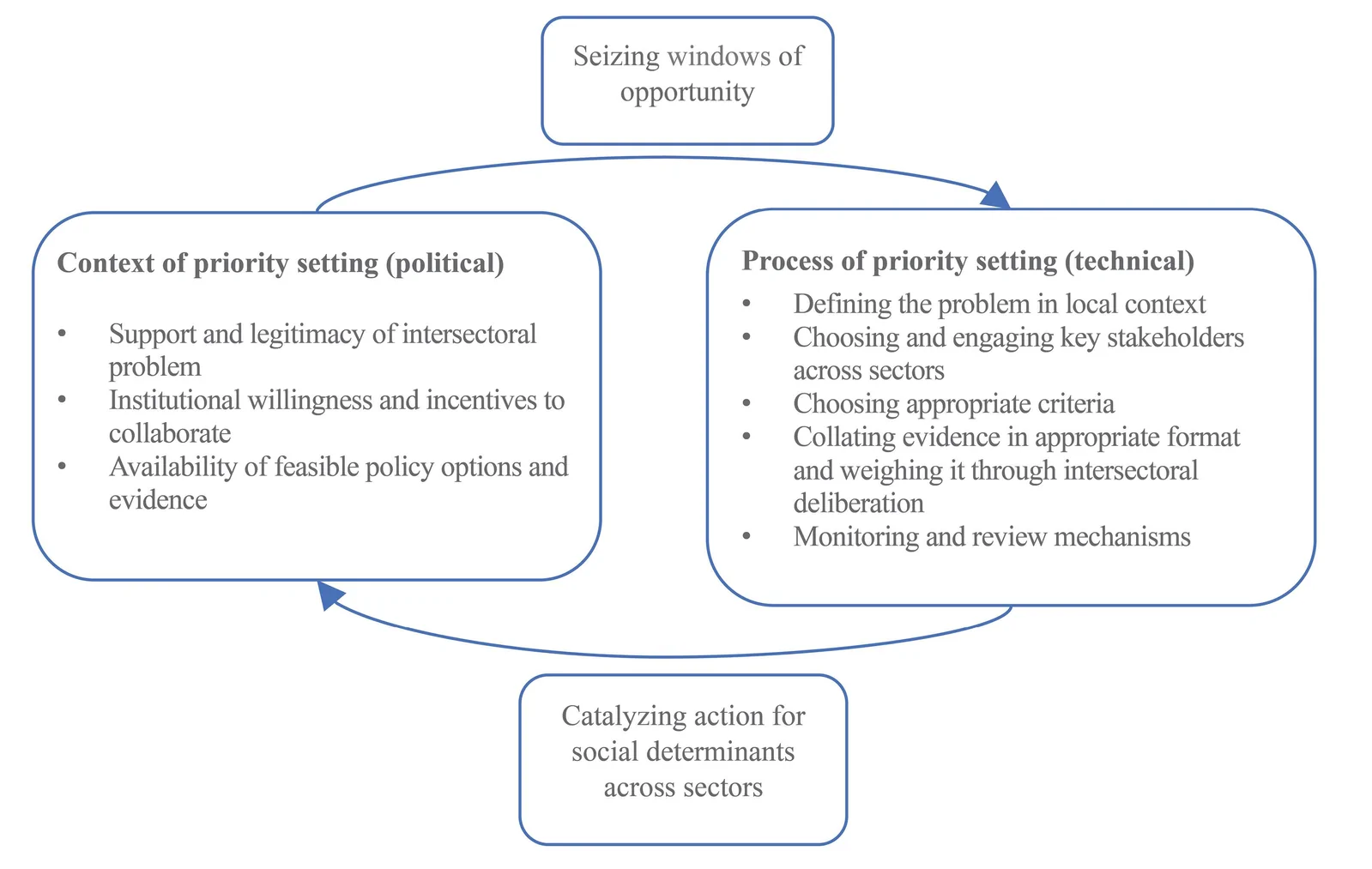
Interaction of Political and Technical Aspects of Intersectoral Priority Setting for Health.

###  Limitations

 We explored a federal perspective from a highly devolved country where provincial health ministries have considerable policy-making powers and often work independently of the federal government. Moreover, we only captured the views of those stakeholders who were closely involved in the priority setting process. Perspectives from other ministries such as finance, trade and commerce and industries would be valuable and likely different. Since ministries are organized differently across governments, the specifics of Pakistan’s case may not fully apply elsewhere. Nevertheless, the core rationing issues are consistent and could be translated to similar contexts. Additionally, the analysis does not explore issues of fiscal policy and budget implications.

## Conclusions

 This study presents the first systematic examination of resource allocation for intersectoral actions for health through the lens of priority setting and agenda setting. Our findings illustrate how a priority setting approach can help health ministries systematize and institutionalize an intersectoral agenda for health. Systematic evidence-informed priority setting could provide a powerful rationale for adopting intersectoral actions as a core health policy agenda that endures beyond tenures of political administrations.

 Our findings also provide insight into institutional factors, such as the lack of established intersectoral decision-making venues, which might derail attempts at instituting such an approach. To advance a prioritized intersectoral agenda effectively, the health ministry not only needs to invest in technical skills for evidence generation and communication but also become a more adept political actor in building alliances based on shared objectives and co-benefits across sectors.

 Interpreting our findings through the combined lenses of priority setting and agenda setting, we propose a novel theoretical approach for organizing, positioning, and advocating priority health-related intersectoral actions in the broader political space across sectors. This approach highlights the dynamic interaction between the technical and political dimensions of priority setting. Using this model, health ministries can use emerging windows of opportunities and proactively generate opportunities to enact their prioritized intersectoral agenda. Finally, our study raises important methodological and normative questions, offering a research agenda to refine and operationalize an intersectoral priority setting approach.

## Acknowledgements

 This research was conducted as part of the Takemi Fellowship at the Harvard School of Public Health. The lead author would like to thank the Takemi program for hosting her as a Takemi Fellow in International Health (2023–2024).

## Disclosure of artificial intelligence (AI) use

 AI was used for language editing in the manuscript.

## Ethical issues

 Ethical approval for the study was received from the National Bioethics Committee for Research in Pakistan with reference number 4-87/NBC-963/22/2135. The project is registered with the Rette in Norway with reference number R4080.

## Conflicts of interest

 Authors declare that they have no conflicts of interest.

## Endnotes


^[1]^ Referred to as the health ministry henceforth.
^[2]^ Referring to federal and provincial ministries.

## Supplementary files


Supplementary file 1 contains Tables S1-S2.
