# Supplementary material for: How Intersectoral Priority Setting Can Support Action on Social Determinants of Health: A Qualitative Case Study From Pakistan’s Federal Health Ministry
Source: Int J Health Policy Manag. 2026 Jul 1;15:9208. doi: 10.34172/ijhpm.9208 (PMC13729801; doi:10.34172/ijhpm.9208)
Supplement: Supplementary file 1 [file ijhpm-15-9208-s001.pdf]

Article title: How Intersectoral Priority Setting Can Support Action on Social Determinants of Health: A Qualitative Case Study From Pakistan's Federal Health Ministry

Journal name: International Journal of Health Policy and Management (IJHPM)

Authors' information: Wafa Aftab[1,2,3]*, Ole F. Norheim[1,4], Inger Lise Teig[1], Sameen Siddiqi[2], Jesse B. Bump[1,3,4]

[1]Bergen Center for Ethics and Priority Setting in Health, Department of Global Public Health and Primary Care, University of Bergen, Bergen, Norway.

[2]Department of Community Health Sciences, Aga Khan University, Karachi, Pakistan.

[3]Takemi Program for International Health, Harvard T.H. Chan School of Public Health, Boston, MA, USA.

[4]Department of Global Health and Population, Harvard T.H. Chan School of Public Health, Boston, MA, USA.

***Correspondence to:** Wafa Aftab; Email: wafa.aftab@uib.no

Citation: Aftab W, Norheim OF, Teig IL, Siddiqi S, Bump JB. How intersectoral priority setting can support action on social determinants of health: a qualitative case study from Pakistan's Federal Health Ministry. Int J Health Policy Manag. 2026;15:9208. doi:10.34172/ijhpm.9208

Supplementary file 1

Supplementary Table 1. Key normative and methodological issues identified in intersectoral priority setting for health

Stakeholder engagement across sectors

- What are the appropriate institutional forums for choosing priority interventions across sectors?
- When existing institutional norms work against collaboration, how can they be shifted?
- What roles should health, other sectors, and overarching ministries play in priority setting and subsequent implementation?

Substantive criteria

- What normative criteria should be used to choose priority interventions (e.g., equity, feasibility, efficiency, etc.)?
- What common objectives, beyond health, can be used across different sectors for a shared process of priority setting?

Choosing appropriate evidence

- What kinds of evidence do policy makers from health and other sectors need to make intersectoral priority setting decisions?

Technical capacities

- What specific capacities must health ministries/institutes of public health invest in to support intersectoral actions?

Supplementary Table 2. Implications of the political context for intersectoral priority setting for across agenda setting domains		
Domain	Key study findings	Implications
Support for and legitimacy of intersectoral actions for health	-Low support for preventive actions<br>-Increase in priority with confluence of some contextual factors	-Proactive role needed by the ministry of health in raising support for identified intersectoral priorities<br>-Need to explore the role of other actors outside the government
Institutional willingness and incentives to collaborate	-Rigid formal sectoral mandates hinder collaboration across sectors disincentivizing formal collaboration	-Engendering high-level political support for a health determinants agenda around shared goals with other sectors.<br>- Human/social development framing rather than only health benefits<br>-Leveraging on existing openness to intersectoral policies across the government for institutional reorganization
Availability of feasible policy options and evidence	-Path dependencies constrain available policy options<br>-Limited evidence available about health and non-health impact of many intersectoral actions<br>-Limited evidence in the local context	-Increase technical competence in health ministry and institutes of public health to produce, collate, and communicate evidence across sectors<br>-Clarify co-benefits of intersectoral policies for other sectors
Windows of opportunities and catalyzing change	-Current focus in health on intersectoral actions reactive and opportunistic<br>-Often dependent on priorities set by other sectors and broader political agendas	-Intervening in the policy cycle at the most opportune points<br><br>-Identifying and leveraging the role of policy entrepreneurs in health and other sectors
